# Mapping TRPM7 Function by NS8593

**DOI:** 10.3390/ijms21197017

**Published:** 2020-09-23

**Authors:** Vladimir Chubanov, Thomas Gudermann

**Affiliations:** Walther-Straub Institute of Pharmacology and Toxicology, Faculty of Medicine, Ludwig-Maximilians Universität München, 80336 Munich, Germany

**Keywords:** TRPM7, NS8593, naltriben, calcium, magnesium, zinc

## Abstract

The transient receptor potential cation channel, subfamily M, member 7 (TRPM7) is a ubiquitously expressed membrane protein, which forms a channel linked to a cytosolic protein kinase. Genetic inactivation of TRPM7 in animal models uncovered the critical role of TRPM7 in early embryonic development, immune responses, and the organismal balance of Zn^2+^, Mg^2+^, and Ca^2+^. TRPM7 emerged as a new therapeutic target because malfunctions of TRPM7 have been associated with anoxic neuronal death, tissue fibrosis, tumour progression, and giant platelet disorder. Recently, several laboratories have identified pharmacological compounds allowing to modulate either channel or kinase activity of TRPM7. Among other small molecules, NS8593 has been defined as a potent negative gating regulator of the TRPM7 channel. Consequently, several groups applied NS8593 to investigate cellular pathways regulated by TRPM7. Here, we summarize the progress in this research area. In particular, two notable milestones have been reached in the assessment of TRPM7 druggability. Firstly, several laboratories demonstrated that NS8593 treatment reliably mirrors prominent phenotypes of cells manipulated by genetic inactivation of TRPM7. Secondly, it has been shown that NS8593 allows us to probe the therapeutic potential of TRPM7 in animal models of human diseases. Collectively, these studies employing NS8593 may serve as a blueprint for the preclinical assessment of TRPM7-targeting drugs.

## 1. Introduction

TRPM7 has been cloned and functionally characterized two decades ago [1,2,3]. Since then, extensive investigations have been conducted to clarify the molecular and organismal aspects of the TRPM7 function [4]. The outcome of these studies has been comprehensively discussed in a number of recent review articles [5,6,7,8,9,10,11]. Here, we briefly highlight the key findings to outline the progress in this fascinating research field.

## 2. Functional Characteristics and Physiological Roles of TRPM7

**Figure 1:**
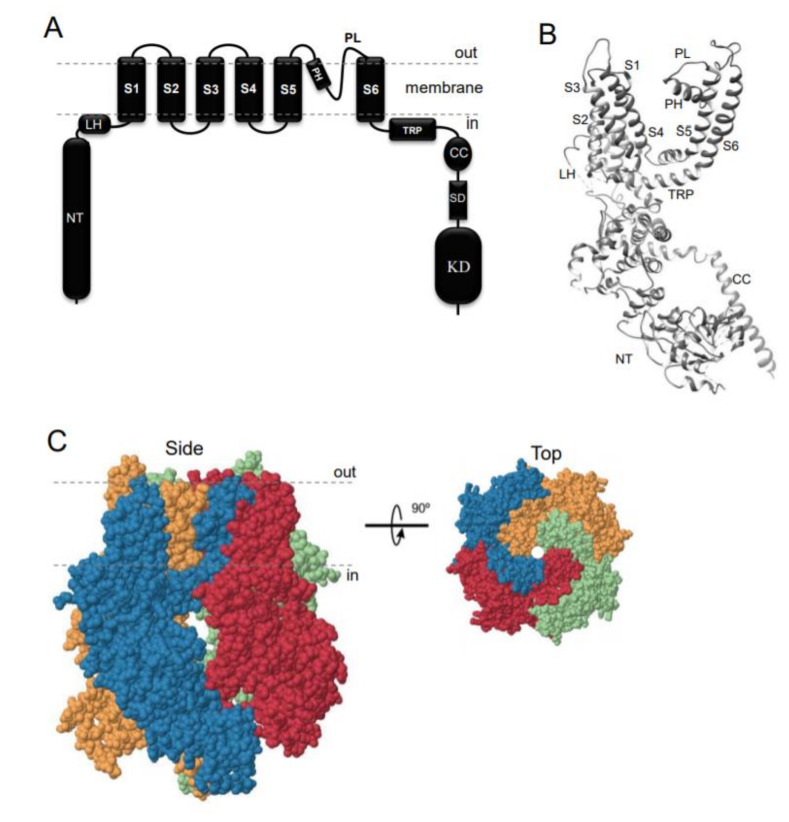
Domain topology and tetrameric assembly of the kinase-coupled channel TRPM7. (**A**) TRPM7 comprises a large cytosolic N-terminal domain (NT), a linker-helical domain (LH), six transmembrane helices (S1-S6), a pore-forming pore helix (PH) and loop (PL), a transient receptor potential domain (TRP), a coiled-coil domain (CC), a kinase substrate domain (SD) and a kinase domain (KD). (**B**) Ribbon diagram of a single TRPM7 channel subunit produced from 6BWD using UCSF Chimera (www.cgl.ucsf.edu). (**C**) Tetrameric TRPM7 channel complex (6BWD) Four channel subunits of TRPM7 are labeled by different colors and shown from the side and top views.

TRPM7 encodes a bi-functional protein comprising a TRP-type transmembrane channel unit fused to a C-terminal α-type serine/threonine-protein kinase domain [1,2,3]. Similarly to other TRP channels, the channel-coding segment of TRPM7 comprises six transmembrane helixes with a channel pore-forming sequence located between the fifth and six helices (Figure 1A,B). Four TRPM7 proteins assemble in a symmetric channel complex (Figure 1C) [1,2,3]. Hence, one TRPM7 channel moiety is linked to four cytosolic kinase domains. Among other known channels and kinases, only TRPM7 and its homologous protein TRPM6 are known as channels covalently fused to protein kinase domains [12,13,14,15]. The crystal structure of the C-terminal TRPM7 domain revealed the three-dimensional packing of the catalytic domain of the kinase [16]. More recently, cryo-electron microscopy of the truncated TRPM7 protein (lacking the kinase domain) clarified the role of distinct amino acid residues for the tetrameric assembly of the channel segment (Figure 1B,C) [17]. However, the positioning of the kinase and channel units relative to each other in the full-length TRPM7 protein, as well as distinct rearrangements in TRPM7 folding during channel gating, remain unknown [18].

In pioneering patch-clamp experiments, endogenous TRPM7 currents were referred to as magnesium nucleotide-regulated metal ion currents (MagNuM) [1,19] and magnesium-inhibited cation currents (MIC) [20], and were later called TRPM7-like or TRPM7 currents [5,6,7,8,9,10,11]. Such native TRPM7 currents were monitored in a large variety of primary isolated cells and stable cell lines [5,6,7,8,9,10,11]. In accord with biophysical experiments, TRPM7 transcripts were found to be abundantly present in all native tissues examined [5,6,7,8,9,10,11]. TRPM7 was defined as a constitutively active cation channel highly selective for divalent cations such as Zn^2+^, Ca^2+^ and Mg^2+^ [1,2,3]. Among other factors, cytosolic magnesium (free Mg^2+^ or in complex Mg·ATP) and the plasma membrane phospholipid phosphatidylinositol-4,5-bisphosphate (PIP_2_) were discovered as prime physiological regulators of TRPM7 [1,2,3,21]. While intracellular Mg^2+^ or Mg·ATP directly act as negative regulators of the channel, receptor-dependent phospholipase C activation, and resultant PIP_2_ depletion indirectly result in TRPM7 inactivation [1,2,3,21].

Genetic disruption of TRPM7 in cultured cells revealed that the TRPM7 channel is key to the homeostatic balance of divalent cations including Zn^2+^, Mg^2+^ and Ca^2+^ [14,22,23,24,25,26], cell motility [27,28,29,30,31,32,33,34], proliferation [1,23,24,35,36,37], differentiation [38,39], Ca^2+^ signaling events [40,41] and an ever growing number of other cellular processes [5,6,7,8,9,10,11]. Pathophysiological implications of TRPM7 are widespread and include anoxic neuronal death [42], hypertension [43,44], neurodegenerative disorders [45,46], tissue fibrosis [47,48,49,50], tumour growth/progression [51,52,53,54,55,56,57,58] and abnormal immune responses [59]. Genetic association studies in humans revealed that point mutations in the *TRPM7* gene cause a giant platelet disorder (macrothrombocytopenia) [60]. Experiments with mice currying a global or tissue-specific null mutation in the *Trpm7* locus showed that TRPM7 is required for early embryonic development [22,61,62,63,64], thymopoiesis [61], morphogenesis of the kidney [63], cardiac rhythmicity and repolarization [65], systemic homeostasis of Zn^2+^, Mg^2+^ and Ca^2+^ [22,66], thrombopoiesis [60], and mast cell degranulation [67].

The list of phosphorylation substrates of the TRPM7 kinase is extensive and surprisingly heterogeneous in terms of possible biochemical pathways affected. Thus, TRPM7 kinase can phosphorylate TRPM6 [68], annexin A1 [69], myosin II isoforms [70], eukaryotic elongation factor-2 kinase (eEF2-k) [71], tropomodulin [72], phospholipase C gamma 2 (PLCγ2) [73], stromal interaction molecule 2 (STIM2) [25], Mothers against decapentaplegic homolog 2 (SMAD2) [59], and Ras homolog family member A (RhoA) [74]. Furthermore, multiple serine/threonine residues positioned in a ‘substrate’ segment of TRPM7 are autophosphorylation targets of the kinase domain [11,75,76,77]. In immune cells, the TRPM7 kinase domain can be cleaved from the channel complex by caspases during Fas-receptor stimulation [35]. Another study reported that the cleaved TRPM7 kinase can be detected in several cell lines and that the released kinase is able to translocate into the cell nucleus to phosphorylate histones [78]. The in vivo relevance of these reactions remains to be verified, because, unlike to the mouse strains with the *Trpm7* null mutation, animals carrying the ‘kinase-dead’ point mutation were found to be fertile, and displayed normal pre- and postnatal development, if maintained under regular conditions [59,66,79,80].

## 3. Drug-like Modulators the Channel and Kinase Activity of TRPM7

In light of the bi-functional nature of TRPM7, there is a growing demand for reliable drug-like molecules allowing for selective and distinct modulation of its channel and kinase moieties. Initially, agents acting as unspecific channel inhibitors, such as spermine [20], ruthenium red [81], trivalent cations [82], SKF-96365 [20] and 2-aminoethyl diphenylborinate (2-APB) [83], were used to block the TRPM7 channel. Subsequently, several drug-like molecules were reported as inhibitors of the TRPM7 channel effective only in a high µM range, such as nafamostat [84], carvacrol [85,86,87,88,89], 5-lipoxygenase inhibitors (NDGA, AA861 and MK886) [90,91,92,93], midazolam [94,95], ginsenoside Rg3 [96], ginsenoside-Rd [97,98], aripiprazole [99] and coomassie brilliant blue G-250 (BBG) [100]. Our laboratory identified several additional inhibitors of the TRPM7 channel such as quinine, CyPPA, dequalinium, SKA31, and UCL1684 [101].

**Figure 2:**
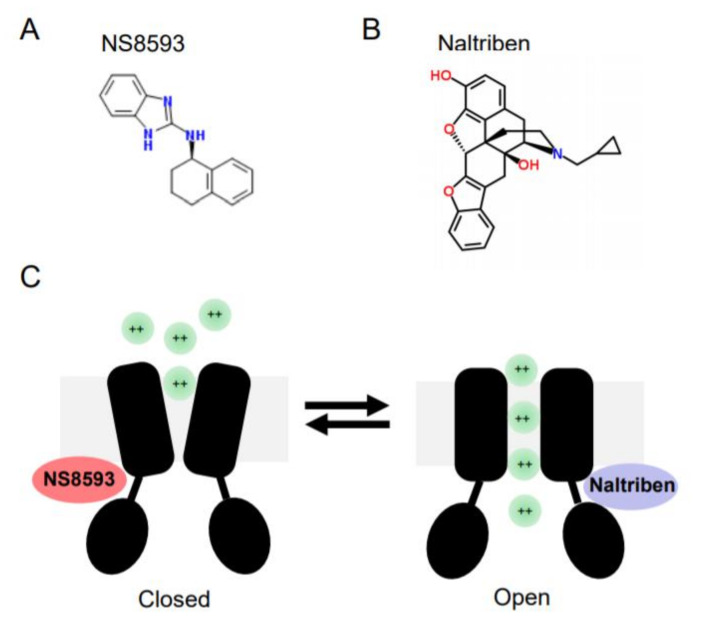
Chemical structures and mode of action of NS8593 and naltriben on the TRPM7 channel. (**A**) NS8593 chemical structure. (**B**) Naltriben chemical structure. (**C**)The TRPM7 channel is depicted in the closed and open states. NS8593 acts as negative gating modulator of the channel, whereas naltiben triggers opening of TRPM7 and influx of divalent cations (green balls) into the cell.

In contrast to later molecules, Waixenicin A, FTY720 and NS8593 were able to suppress TRPM7 currents when applied at low µM concentrations. Subsequently, these reagents were often used to probe the cellular role of TRPM7 [102,103]. Waixenicin A is a natural terpenoid isolated from the soft coral *Sarcothelia edmondsoni*, and inactivates the TRPM7 channel in an Mg^2+^ dependent manner with an IC_50_ of 7 µM [37]. FTY720 (synthetic homolog of sphingosine) inhibited TRPM7 currents with an IC_50_ 0.7 µM [104]. Our laboratory has shown that the small synthetic molecule N-[(1R)-1,2,3,4-tetrahydronaphthalen-1-yl]-1H-benzimidazol-2-amine (NS8593, Figure 2) suppresses TRPM7 currents in an Mg^2+^-dependent fashion with an IC_50_ of 1.6 µM [101].

In a follow-up screen, our group has discovered the first small drug-like molecules functioning as TRPM7 channel agonists [105]. In particular, we found that twenty drug-like compounds with different structural backbones can stimulate TRPM7 currents [105,106]. Among them, naltriben (Figure 2) and mibefradil were characterized more in detail, and nowadays both compounds are frequently used by other TRPM7 investigators, often in combination with the TRPM7 inhibitors mentioned before [102,103]. Of note, naltriben is able to activate the TRPM7 channel both in the presence of physiological concentrations of cytosolic Mg^2+^ and after PIP_2_ depletion with an EC_50_ of 20 µM [105]. Hence, we defined naltriben as a positive gating modulator of the TRPM7 channel [105]. Unlike naltriben, mibefradil-mediated activation of TRPM7 was highly dependent on intracellular Mg^2+^ levels [106]. Accordingly, we suggested that at least two distinct types of TRPM7 activators exist, referred to as type 1 (acting independently of Mg^2+^) and type 2 (Mg^2+^-dependent agonists) [106].

Overall, the pharmacological toolkit suitable for the assessment of the TRPM7 kinase remains underdeveloped, and currently, it is limited to only one compound, TG100-115. TG100-115 was initially introduced as an inhibitor of phosphoinositide 3-kinases [107]. However, Davis et al. [108] found that TG100-115 is also able to inactivate the purified kinase domain of TRPM6 with an IC_50_ of 8 nM [108]. We also found that TG100-115 efficiently inactivates TRPM6 kinase in living cells [21]. Besides, Song et al. [109] examined the effects of TG100-115 on the TRPM7 kinase and reported that this reagent inhibits the TRPM7 kinase with an IC_50_ of 2 µM. Finally, it is worth mentioning that in our hands, neither NS8593 nor naltriben directly affected the kinase activity of TRPM7 (unpublished observations). However, we cannot rigorously exclude that in specific experimental settings, these compounds may modulate the kinase moiety indirectly, for instance subsequent to altered uptake of divalent cations by the channel domain of TRPM7.

## 4. NS8593 as a Tool to Investigate the Function of TRPM7 Currents

As mentioned above, the small synthetic compound NS8593 was identified as a potent inhibitor of the TRPM7 channel [101]. In recent years, numerous independent studies have been conducted, and NS8593 was successfully used to probe the role of TRPM7 in various cellular processes (Table 1). Overall, these results have made significant inroads into our understanding of the druggability of the TRPM7 channel and highlighted several pathophysiological conditions, which can be modulated by TRPM7 inhibitors.

Initially, NS8593 was identified as a potent negative gating modulator of small conductance Ca^2+^-activated K^+^ channels (SK1-3 or K_ca_2.1-2.3 channels) [123]. The inhibitory effect of NS8593 was pronounced at low intracellular Ca^2+^ concentrations and abolished at 30 μM Ca^2+^ [123]. It turned out that NS8593 is also able to suppress TRPM7 currents in an analogous manner [101]. Thus, the effect of NS8593 on TRPM7 is modulated by cytosolic Mg^2+^ levels, because the IC_50_ value of NS8593 determined in the absence of Mg^2+^ (1.6 µM) was increased ~4-fold in the presence of 300 μM Mg^2+^ [101]. The inhibition of TRPM7 currents by NS8593 was voltage-independent. Importantly, the effect of NS8593 was fast, reversible, and repeatable, suggesting that the interaction of NS8593 with TRPM7 neither induces irreversible modifications of the protein nor affects the cell surface localization of TRPM7 [101]. Patch-clamp experiments with a subset of primary cell models (freshly isolated smooth muscle cells, primary mouse podocytes, and primary human ventricular myocytes) demonstrated that NS8593 efficiently blocks endogenous TRPM7 currents [101]. Notably, a highly specific inhibitor of SK channels, apamin, showed no effect on the biophysical characteristics of TRPM7 and can, therefore, be used for sorting-out off-target effects of NS8593 in cells expressing both channel species [101]. Finally, long-term exposure of cultured cells to NS8593 showed that this compound elicits sustained effects on TRPM7 [101]. For instance, the addition of NS8593 (but not apamin) to the cell culture medium suppressed endogenous TRPM7 currents and motility of HEK293 cells, replicating one of the prominent effects of siRNA silencing of TRPM7 [101]. Collectively, these findings suggested that NS8593 acts as a negative gating modulator of the TRPM7 channel (Figure 2B), and that NS8593 is well suitable for blocking endogenous TRPM7 channels in different experimental settings.

In follow up studies, many researchers used NS8593, frequently in combination with other TRPM7 modulators or RNAi silencing, to characterize the role of TRPM7 in particular cellular pathways (Table 1). For example, Davis et al. [120] employed NS8593 to study the mechanisms of epithelial-mesenchymal transition (EMT) of MDA-MB-468 breast cancer cells. The authors conducted siRNA-based screens and identified the TRPM7 channel as a critical regulator of epidermal growth factor (EGF)- or hypoxia-induced STAT3 phosphorylation and of the expression of the EMT marker vimentin in a Ca^2+^-dependent fashion. Accordingly, siRNA silencing of TRPM7 or application of NS8593 suppressed EGF-induced EMT in MDA-MB-468 cells [120]. Tumour necrosis factor-related apoptosis-inducing ligand (TRAIL) can selectively induce apoptosis in various types of cancer cells. Song et al. [124] investigated whether TRPM7 knockdown or pharmacological inhibition of the channel can enhance TRAIL-induced apoptosis in triple-negative breast cancer cells (MDA-MB-231 and MDA-MB-468). Among other findings, the authors demonstrated that pharmacological inhibition of the channel or kinase units in TRPM7 using NS8593 and TG100-115, respectively, synergistically increases TRAIL-induced apoptosis in breast cancer cells [124].

Among the critical microglial responses to brain injury is the activation and migration of microglia cells to the sites of injury. Lipopolysaccharide (LPS)- and interleukin-induced microglial activation is associated with characteristic changes in transcriptional profiles, production of inflammatory mediators, and increased motility of microglial cells. Siddiqui et al. [119] used NS8593 to illustrate that TRPM7 is required for interleukin-4 and -10 (IL-4, -10) induced motility of primary rat microglial cells. These findings are in line with the study of Schilling et al. [110], who demonstrated that TRPM7 inhibitors NS8593 or FTY720 suppressed proliferation of bone-marrow-derived macrophages induced by IL-4 and macrophage colony-stimulating factor (M-CSF). In addition, NS8593 and FTY720 prevented polarisation of primary macrophages towards the anti-inflammatory phenotype [110].

Nörenberg et al. [100] used NS8593 to show that TRPM7 regulates ATP-induced currents, which were previously thought to be conducted by the P2X7 channel. P2X7 mediates nonselective cation currents that are typically elicited by high concentrations of extracellular ATP. Nörenberg et al. [100] re-examined such ATP-induced currents in HEK293 and rat glioma C6 cells and concluded that TRPM7 is the correct molecular correlate of ATP-induced currents. In another study, Sadowska et al. employed NS8593 to rule out the involvement of TRPM7 in Ca^2+^-dependent osmosensing of nucleus pulposus cells [125].

Krishnamoorthy et al. [121] took advantage of NS8593 to demonstrate that TRPM7 controls antigen internalization and presentation in B cells. The DT40 B cell line deficient in the TRPM7 gene was unable to aggregate antigen after activation likely due to abnormal phospholipase C (isoform γ2) signalling and altered lipid metabolism. These results were recapitulated in primary mouse B cells expressing only a single allele of *Trpm7* or after treatment by NS8593. The authors suggested TRPM7 controls an essential process required for B cell affinity maturation and the production of high-affinity antibodies [121].

Prostaglandin E2 (PGE2) plays a role in the migration and proliferation of human glioblastoma cells [111]. Tian et al. [111] observed that PGE2 increased TRPM7 currents in human glioblastoma A172 cells. Knockdown of TRPM7 by shRNA or exposure of cells to NS8593 abrogated PGE2-stimulated motility and proliferation of A172 cells [111]. Zou et al. [114] used NS8593 as one of the tools to establish an interplay between TRPM7 and epidermal growth factor receptor (EGFR) signalling in vascular smooth muscle (VSMC) cells. The study was conducted in primary VSMCs from rats and humans treated by NS8593 or cells from vascular tissues of *Trpm7*-modified mice. Zou et al. [114] found that EGFR directly interacts with TRPM7. This interaction regulated cytosolic Mg^2+^ levels, ERK1/2 signalling, and vascular tissue homeostasis.

*Plasmodium falciparum* causes the most harmful form of malaria in humans. The parasite invades erythrocytes and triggers complex responses due to multiple ligand-receptor interactions leading to the abnormal assembly of cytoskeletal proteins [122]. Intriguingly, Sisquella et al. [122] have shown that NS8593, as well as other TRPM7 blockers (FTY720 and waixenicin), fully inhibit parasite invasion and changes in deformability of erythrocytes suggesting that TRPM7 might be a promising target of new antimalarial drugs.

Independent studies with TRPM7 gene-deficient cells revealed a critical role of TRPM7 in the cellular balance of Mg^2+^ [14,22,23,24,25,26]. In line with this idea, several laboratories could show that NS8593 interferes with TRPM7-mediated uptake of Mg^2+^. For instance, Tashiro et al. [112,113] employed NS8593 in combination with naltriben to illustrate that TRPM7 controls Mg^2+^ influx in primary rat ventricular myocytes. Thus, endogenous TRPM7 currents were abundantly present in primary cells and were fully blocked by NS8593. In line with these findings, application of NS8593 lowered levels of cytosolic Mg^2+^. The authors also noted that naltriben significantly raised cellular levels of Mg^2+^ after the removal of extracellular Na^+^ to offset the activity of Na^+^/Mg^2+^ exchangers. However, re-introduction of extracellular Na^+^ lowered Mg^2+^ concentrations to the basal level. The authors concluded that Mg^2+^ entry through TRPM7 significantly contributes to Mg^2+^ homeostasis in mammalian heart cells [112,113]. In another study, Luongo at al. [115] used NS8593 to illustrate such a role of TRPM7 in human epithelial colon cells. Interestingly, colon carcinoma HT29 and HCT116 cells express the two homologous proteins TRPM6 and TRPM7. NS8593 treatment or TRPM7 silencing by RNAi suppressed cell proliferation and Mg^2+^ influx in both HT29 and HCT116 cells, while downregulation of TRPM6 did not significantly affect either Mg^2+^ influx or cell proliferation [115].

NS8593 was also instrumental in studies investigating the contribution of TRPM7 to Ca^2+^ signalling events. It is well established that repetitive oscillations in cytoplasmic Ca^2+^ due to periodic influx of Ca^2+^ drive mammalian embryo development following fertilization. Carvacho et al. [41] and Bernhardt et al. [116] focused on the identification of channels controlling such mechanisms. Thus, Carvacho et al. [41] detected TRPM7 currents in immature mouse oocytes (germinal vesicle stage), matured oocytes (metaphase II eggs) and 2-cell stage embryos. Currents were activated by natriben and inhibited by NS8593. Activation of TRPM7 induced Ca^2+^ influx in oocytes and eggs to support fertilization and egg activation. Application of NS8593 delayed pre-implantation embryo development and reduced progression to the blastocyst stage. This concept was further supported by the study of Bernhardt et al. [116] showing that fertilization-induced Ca^2+^ oscillations in mouse oocytes and eggs were also impaired by NS8593 treatment. Hence, both studies suggest that TRPM7 may contribute to Ca^2+^ influx in post-fertilization oocytes, eggs, and in embryonic development in mice.

In various cells, Ca^2+^ release from the endoplasmic reticulum engages calcium release-activated calcium channels (CRAC), a process that is entitled as store-operated calcium entry (SOCE). Two recent studies reviled that TRPM7 may be functionally connected to these pathways. Faouzi et al. [25] found that TRPM7 gene-deficient DT40 B lymphocytes exhibit impaired SOCE. In accord, blockade of TRPM7 with NS8593 or waixenicin A in wild-type cells results in a reduced SOCE. Using DT40 cells expressing a kinase-deficient mutant of TRPM7, Faouzi et al. [25] showed that TRPM7 regulates SOCE through its kinase domain. In line with these findings, NS8593 in combination with naltriben and siRNA approach was instrumental in demonstrating that in rat primary enamel cells and murine ameloblast LS8 cells TRPM7 acts as a positive regulator of SOCE and that this function of TRPM7 is dependent on ORAI1/2 channels, known molecular correlates of SOCE [117].

In adipocytes, cytosolic Ca^2+^ regulates insulin responses and the secretion of adipokines. Inoue et al. [118] investigated whether TRPM7 contributes to Ca^2+^ influx in freshly isolated white adipocytes and in 3T3-L1 adipocytes differentiated from 3T3-L1 pre-adipocyte cells. The authors used NS8593 together with FTY720, naltriben and siRNA techniques to show that the TRPM7 channel is functionally expressed in adipocytes. The authors conclude that TRPM7 plays a role as a Ca^2+^ influx pathway in adipocytes [118].

## 5. Assessment of NS8593 Effects in Animal Models

Originally, NS8593 was described as the selective blocker of SK channels, which are abundantly expressed in the heart and considered as a new therapeutic target for the treatment of atrial fibrillation (AF). Accordingly, NS8593 was tested in several ex vivo and in vivo models of AF. For example, in a rat model of AF, injection of NS8593 (5 mg/kg) shortened AF duration equally to amiodarone (known anti-AF drug) [126]. Similarly, injection of NS8593 (5 mg/kg) was found to be beneficial in AF models in dogs and horses [127,128]. While the specific contribution of SK channels vs. TRPM7 in such anti-AF effects remains to be elucidated [129,130], these studies clearly showed that living animals could well tolerate the systemic administration of NS8593.

More recently, NS8593 was used to assess TRPM7 as a new anti-fibrotic pharmacological target (Table 1). Expression of TRPM7 was found to be upregulated in fibrotic tissues of lung, liver and heart fibrosis [50,131,132,133,134]. TRPM7 expression was also increased after renal ischemia-reperfusion leading to kidney injury and fibrosis [135,136]. Recently, Suzuki et al. [49] investigated the unilateral ureteral obstruction (UUO) mouse model and observed that TRPM7 expression was elevated in UUO kidneys. Intraperitoneal injection of NS8593 (7 days; 5 mg/kg/day) prevented kidney atrophy in UUO kidneys, retained tubular formation, and reduced TRPM7 expression to normal levels. Mechanistically, the authors suggested that TRPM7 affects tissue fibrosis via the TGF-β1/Smad network. The authors propose that pharmacological targeting of TRPM7 may be used to suppress kidney fibrosis [49].

As discussed above, TRPM7 inhibitors showed anti-proliferative effects on many cultured tumour-derived cells. Recently, our laboratory explored hepatocellular carcinoma HuH7 cells in a xenograft mouse model to assess the efficiency of NS8593 to suppress tumour progression (Table 1) [74]. Thus, we treated nude mice bearing xenografts derived from HuH7 cells systemically by intravenous injection of NS85936 (6 mg/kg every 2nd day, 17 days) and observed profoundly reduced tumour growth in NS85936-treated mice when compared with control animals [74]. Further experiments with multiple cell models revealed that the anti-tumour effect of NS8593 relies on TRPM7 channel-mediated Mg^2+^ influx and phosphorylation of RhoA by TRPM7 kinase [74].

## 6. Conclusions

Several structurally unrelated pharmacological modulators of TRPM7 have been identified, including NS8593—a commercially available potent inhibitor of the TRPM7 channel. NS8593 was found to be instrumental in a broad range of experimental settings such as transient inactivation of TRPM7 currents in patch-clamp measurements, sustained treatment of cultured cells, and administration of the compound to living mice. Pharmacological targeting of TRPM7 by NS8593 in conjunction with genetic silencing of the whole TRPM7 protein or comparative analysis of effects induced by structurally unrelated TRPM7 modulators were shown to be instrumental in uncovering new cellular functions of TRPM7 and assessing the therapeutic potential of anti-TRPM7 drugs. Accordingly, these experiments can be regarded as a blueprint for the further development of high-affinity in vivo drugs acting on TRPM7.

## Figures and Tables

**Table 1:** Cellular processes affected by NS8593.

Cell Type/Tissue	Effects of NS8593	References
HEK293,	Motility/chemotaxis	[101]
primary microglia,		[110]
A172 glioblastoma cells		[111]
Primary ventricular myocytes,	Mg^2+^ homeostasis	[112,113]
primary vascular smooth muscle cells,		[114]
HT29 epithelial colon cells		[115]
Mouse oocytes and eggs,	Ca^2+^ uptake, intracellular Ca^2+^ stores and Ca^2+^ signalling	[41,116]
DT40 B lymphocytes,		[117]
primary enamel cells,		[118]
3T3-L1 fat cells		[119]
MDA-MB-231 breast cancer cells,	Proliferation/cell cycle	[120]
A172 glioblastoma cells		[111]
MDA-MB-468 breast cancer cells	Differentiation	[121]
Primary B cells,	Immune responses	[122]
mouse erythrocytes		[123]
primary microglia		[120]
primary macrophages		[110]
HuH7 hepatocellular carcinoma in a xenograft mouse model	Tumour growth	[74]
Kidneys	Fibrosis	[49]

## Abbreviations

AF	atrial fibrillation
EGF	epidermal growth factor
EGFR	epidermal growth factor receptor
EMT	epithelial-mesenchymal transition
IL	interleukins
LPS	lipopolysaccharides
PGE2	prostaglandin E2
PIP2	phosphatidylinositol-4,5-bisphosphate
PLCγ2	phospholipase C gamma 2
RhoA	Ras homolog family member A
SK channels	small conductance Ca^2+^-activated K^+^ channels
SOCE	store-operated calcium entry
TRPM6	transient receptor potential cation channel, subfamily M, member 6
TRPM7	transient receptor potential cation channel, subfamily M, member 7
UUO	unilateral ureteral obstruction
VSMC	vascular smooth muscle
